# Microbial and metabolic responses of maize silage to leaf blight: implications for fermentation and protein stability

**DOI:** 10.1186/s12870-026-08142-4

**Published:** 2026-01-21

**Authors:** Liuxing Xu, Xianfu Lv, Xiaolu Lu, Xiaolong Zhang, Jianjun Liu, Yuanyan Meng, Dan Wu

**Affiliations:** 1 College of Agronomy and Life Sciences, Zhaotong University, Zhaotong, 657000 China; 2 School of Geographical Sciences and Tourism, Zhaotong University, Zhaotong, Yunnan 657000 China

**Keywords:** Disease, Silage, Lactiplantibacillus plantarum, Microbiome –metabolome interaction, Proteases

## Abstract

Foliar fungal diseases lead to a significant reduction in dry matter of plants, thereby negatively affecting silage fermentation kinetics. This study investigated the effects of leaf blight on ensiling kinetics, microbial succession, and nitrogen metabolism in whole-crop maize, and further examined whether inoculation with *Lactiplantibacillus plantarum* HT1 could mitigate disease-induced proteolysis through remodeling of the metabolic pathways. Three treatments were established: (i) healthy maize silage (CON), (ii) maize silage affected by leaf blight (DCON), and (iii) maize silage affected by leaf blight and inoculated with *L. plantarum* HT1 (HT1, 1 × 10^5^ cfu/g FM based on the fresh weight basis). Samples of 300 g fresh material were packed into polyethylene vacuum bags (300 × 400 × 0.2 mm) and vacuum-sealed to establish anaerobic conditions for 60 days of ensiling. Before ensiling, DCON had significantly lower crude protein content (7.06% vs. 8.91% DM, *P* = 0.001) compared with CON. In addition, the WSC content markedly decreased by leaf blight (88.7 vs. 119 g/kg DM, *P* = 0.016). LAB abundance was significantly lower (3.22 vs. 4.22 log10 CFU/g FM, *P* = 0.022), whereas mold counts sharply increased (4.25 vs. 3.22 log10 CFU/g FM, *P* = 0.001) in DCON than in CON. The DCON showed elevated pH, significantly reduced lactic acid content, and markedly increased butyric acid content compared with those of the CON (*P* < 0.05). DCON had the highest NH_3_–N content (17.2 g/kg TN), exceeding those of CON (12.3 g/kg TN) and HT1 (10.3 g/kg TN, *P* = 0.005). Aminopeptidase and carboxypeptidase activities increased to 46.4 and 167 U·h^− 1^·g FM^− 1^, respectively, which were much higher than those of the CON (27.4 and 140 U·h^− 1^·g FM^− 1^). Microbiome β-diversity separated DCON from CON and HT1, with enrichment of putative proteolytic taxa. Metabolomics indicated upregulated amino acid degradation (branched chain and glutamate pathways) and disrupted nitrogen homeostasis in the DCON treatment. Leaf blight created an early high-pH window that amplified proteolysis and nitrogen loss through coordinated shifts in substrates, microbiota, and amino acid catabolism. Inoculation with *L. plantarum* HT1 caused rapid acidification, curtailed proteolysis, and rewired metabolic and community networks toward a healthy state, achieving coordinated restoration of fermentation quality and protein preservation in disease-challenged maize.

## Introduction

Maize (*Zea mays*) is an important forage crop for silage in modern animal husbandry [1, 2]. However, foliar fungal diseases, such as leaf blight, cause leaf necrosis and a decline in photosynthetic capacity [3, 4], resulting in a significant reduction in dry matter (DM) yield per unit area. Leaf blight can reduce maize DM yield by 10–30% while significantly decreasing the contents of water-soluble carbohydrates (WSC) and nitrogen-free extract [5], thereby lowering total digestible nutrient levels and digestible energy.

The fate of proteins during silage fermentation directly determines the feeding value of the silage [6, 7]. Both endogenous plant- and microbial-derived proteases synergistically act under anaerobic conditions, leading to the degradation of crude proteins into peptides, free amino acids, and NH_3_-N [8, 9]. Excessive hydrolysis reduces biological utilization of proteins and results in the accumulation of biogenic amines (e.g., histamine and putrescine) [10], which may pose a risk to animal health [11]. An increased proportion of NH_3_-N in silage is generally regarded as a sign of severe protein degradation [12–14]. Under disease conditions, leaf tissue damage accelerates the release of cellular contents, including nitrogenous compounds [15, 16], and diseases alter the phyllosphere [17] and endophytic [18, 19] microbial communities, further accelerating the rate of protein degradation and increasing the risk of NH_3_‑N accumulation. To mitigate this unfavorable degradation process, lactic acid bacteria (LAB) inoculants are widely used in silage production [20, 21], particularly *Lactiplantibacillus plantarum* [22, 23]. *L. plantarum* can rapidly consume WSC in the early stage of ensiling, produce large amounts of lactic acid and lower the pH, thereby effectively suppressing undesirable microbes and protease activity and reducing the accumulation of NH_3_-N and biogenic amines [24, 25]. *L. plantarum* inoculation reshapes the bacterial community structure, allowing LAB to dominate during the mid-to-late fermentation stages [26] while simultaneously modulating amino acid metabolism, organic acid biosynthesis, and nitrogen metabolic pathways [27, 28]. For instance, 600–900 metabolites can be identified in maize silage, with inoculation treatments showing significant differences in the accumulation of amino acids, phenolic acids, and organic acid metabolites, and these dynamic changes strongly correlate with LAB abundance [29, 30]. However, the impact of fungal diseases, such as leaf blight, on protein degradation patterns, nitrogen flux, and metabolic networks during silage fermentation remains poorly understood.

Previous studies have separately investigated the effects of diseases on raw materials [31], role of LAB inoculants, and associations between microbial communities and metabolism [32]; however, systematic studies using “field disease (mainly leaf blight) as the initial stress” and tracing the entire chain, from chemical properties of raw materials and fermentation kinetics to microbial community succession, metabolite changes, protein degradation, and nitrogenous metabolite accumulation, remain scarce. Therefore, the present study systematically investigates the coupled “disease–microbe–metabolite–protein degradation” mechanisms. This study aims to address three key scientific questions: (1) To what extent does leaf blight-induced physiological deterioration (e.g., substrate depletion and tissue damage) pre-calculate the shift from homolactic to undesirable fermentation pathways?. (2) Through what metabolic nodes and microbial drivers does the disease-driven dysbiosis coordinate to accelerate nitrogen flux toward excessive proteolysis? (3) Can *L. plantarum* HT1 effectively stabilize the protein fraction by neutralizing the “early high-pH window” and remodeling the metabolic networks under disease stress?

## Materials and methods

### Experimental design and treatments

A randomized block design with three biological replicates per treatment was used. Three treatments were established: (i) healthy maize silage (CON), (ii) maize silage affected by leaf blight (DCON), and (iii) leaf blight–affected maize silage inoculated with *L. plantarum* HT1 (HT1). All maize plants were collected from the same field and harvested at the milk stage (Biologische Bundesanstalt, Bundessortenamt and Chemical Industry 75) to ensure uniform maturity. Maize was sown on May 2, 2022 and harvested on September 22, 2022 in the experimental field of Zhaotong University, Guoxue Road, Zhaoyang District, Zhaotong City, Yunnan Province, China (27º36′N, 103º74′E; 1985-m altitude). No herbicides or pesticides were applied during the growing period. After harvesting, all whole plants were immediately transported to the laboratory, where large impurities were removed. Tissues were then meticulously sorted into “healthy” and “diseased” groups based on typical visual symptoms, specifically characteristic cigar-shaped necrotic lesions (gray-green to tan) on the leaves, with uniform disease severity selected to ensure consistency. After sorting, the plants were chopped into 2–3 cm pieces using a laboratory-scale electric forage cutter (Model 9Z-0.4; Zhengzhou Azeus Machinery, Henan, China) and thoroughly mixed. Subsamples of 300 g fresh material were packed into polyethylene vacuum bags (300 × 400 × 0.2 mm; Mingkang Packing, Zhongshan, China) and vacuum-sealed (Sinbo Vacuum Sealer; Hong Tai Home Electrical Co., Hong Kong, China) to establish anaerobic conditions. The inoculant strain *L. plantarum* HT1 (provided by the Forage Processing Laboratory, South China Agricultural University) was applied at 1 × 10^5^ colony-forming unit (CFU)/g fresh matter (FM). For the HT1 treatment, the bacterial suspension was evenly sprayed onto the chopped material, followed by 2–3 rounds of thorough mixing to ensure uniform distribution. The CON and DCON were administered the same volume of sterile physiological saline. All vacuum-sealed silage bags were stored at ambient temperature (17–25 °C). At the end of the storage period, the silage samples were opened and analyzed for fermentation quality, microbial community composition, and metabolite profiles. At the end of the 60-day storage period, all silage bags were opened. The contents were thoroughly mixed, and subsamples were partitioned for distinct analyses. Specifically, one portion (20 g) was used for preparing aqueous extracts (1:4 ratio with distilled water) to determine pH and organic acids. Another portion (10 g) was immediately used for microbial plate counting. For multi-omics analysis, 50 g of material was snap-frozen in liquid nitrogen to preserve the microbial and metabolic state. To ensure representative results, all sampling was performed rapidly to minimize exposure to air.

## Analysis of chemical, microbial, and silage fermentation quality

The DM content was determined by drying the samples at 65 °C to a constant weight and was calculated based on fresh matter. The crude protein content was determined using the Kjeldahl method for total nitrogen (KN680; Shandong Jinan Alva Instrument, Jinan, China). Neutral (NDF) and acid (ADF) detergent fiber contents were measured using the filter bag technique [33], in which the samples were extracted with neutral and acid detergent solutions, respectively, followed by filtration, drying, and calculation of the fiber content. The WSC content was determined by the anthrone colorimetric method and quantified against a glucose standard curve [34]. pH was measured by suspending the samples in distilled water at a ratio of 1:4 (w/v), soaking at 4 °C for 18 h, and directly analyzing with a pre-calibrated electrode pH meter (LE438 pH meter; Mettler Toledo, Shanghai, China). The buffering capacity was calculated according to the amount of standard acid solution consumed during titration [35], and the results are expressed as mE/kg DM.

Microbial counts were determined by the plate count method [36]. Samples were serially diluted 10-fold with sterile physiological saline and plated on selective media; LAB were incubated anaerobically on de Man, Rogosa, and Sharpe agar for 2–3 days (achieved by sealing plates in vacuum-packed silage bags); aerobic bacteria were incubated on nutrient agar for 1–2 days; and yeasts and molds were incubated on potato dextrose agar for 2–3 days (Guangdong HuanKai Microbial Science & Tech. Co., Ltd., Guangzhou, China). All incubations were conducted at 37 °C, and results are expressed as log10-transformed CFU/g FM.

Organic acids were quantified using high-performance liquid chromatography (HPLC, Agilent 1260; Agilent Technologies Inc., Santa Clara, CA, USA). Separation was performed using a Sodex RS Pak KC-811 column (Showa Denko, Kawasaki, Japan) equipped with a diode array detector (DAD, 210 nm; SPD-20 A; Shimadzu, Kyoto, Japan). The mobile phase comprised 3 mmol/L perchloric acid at a flow rate of 1.0 mL/min, with the column temperature maintained at 60 °C.

Free amino acid nitrogen (FAA-N) content was determined by the ninhydrin colorimetric method and calculated against a glycine standard curve, and NH_3_-N was determined by the phenol–hypochlorite colorimetric method [37] and expressed as a proportion of total nitrogen (g/kg TN). Ninhydrin was used as a developer to measure the free amino acid content [38].

Phosphate buffer (50 mM, pH 7.0) was used as the basic extraction medium, supplemented with 0.15 M NaCl to enhance protein solubility and 2 mM dithiothreitol to maintain the thiol groups of cysteine proteases in a reduced state. Acid proteases were extracted with 50 mM sodium acetate buffer (pH 5.0), and serine proteases were extracted with Tris-HCl buffer (pH 7.5) containing 5 mM CaCl_2_ to improve structural stability [39]. Because aminopeptidases [39] and carboxypeptidases [40] are mostly metal-dependent enzymes, ethylenediaminetetraacetic acid and other metal chelators were avoided during extraction to prevent the inhibition of their activity. After homogenization in the corresponding buffers, the samples were kept at 4 °C for 10 min to facilitate protein solubilization and then centrifuged at 12,000 × *g* for 15 min at 4 °C to remove cell debris and insoluble material. The resulting supernatant was collected as the crude enzyme extract. Aspartate, serine, and cysteine protease activities were determined using enzyme-linked immunosorbent assay kits (Shanghai Chutai Biotechnology Co., Ltd., Shanghai, China), according to the manufacturer’s instructions.

## Analysis of bacterial community structure

The bacterial community was analyzed by amplifying the V3–V4 hypervariable regions of the 16S rRNA gene with the primer pairs 338 F (5′-ACTCCTACGGGAGGCAGCAG-3′) and 806R (5′-GGACTACHVGGGTWTCTAAT-3′). Amplification by polymerase chain reaction (PCR) was performed in a 20-µL reaction volume containing 4 µL of 5× FastPfu buffer, 2 µL of 2.5 mM dNTPs, 0.8 µL of each primer (5 µM), 0.4 µL of FastPfu DNA polymerase, and 10 ng template DNA, with nuclease-free water added for volume make up [41]. The PCR program comprised an initial denaturation at 95 °C for 3 min; 27 cycles of denaturation at 95 °C for 30 s, annealing at 55 °C for 30 s, and extension at 72 °C for 45 s; and a final extension at 72 °C for 10 min. PCR products were visualized using 2% agarose gel electrophoresis, purified using a DNA purification kit (YuHua, Shanghai, China), and quantified using a Qubit 4.0 fluorometer (Thermo Fisher Scientific, USA). Purified amplicons were pooled at equimolar concentrations and subjected to paired-end sequencing on an Illumina NextSeq 2000 platform (Illumina, San Diego, CA, USA) at Majorbio Bio-Pharm Technology Co., Ltd. (Shanghai, China).

Raw sequencing data were processed using a standardized bioinformatics pipeline. Quality control was performed using fastp v.0.19.6 to remove low-quality and short reads, and those containing ambiguous bases. To reduce the impact of sequencing-depth variation on subsequent diversity analyses, all samples were rarefied to 20,000 sequences, with an average Good’s coverage of 99.09%. Representative OTU sequences were taxonomically assigned using RDP Classifier v.2.2 against the Silva 16S rRNA gene database v.138, with a confidence threshold of 70%.

The data were analyzed using the Majorbio Cloud Platform (https://cloud.majorbio.com). Beta diversity was assessed using principal coordinates analysis (PCoA) based on Bray–Curtis distances, and significance of treatment differences was evaluated using permutational multivariate analysis of variance. Differentially abundant taxa among treatments were identified using linear discriminant analysis (LDA) effect size (LEfSe) with an LDA score > 2.0 and *P* < 0.05. The relationships between environmental factors and community structure were examined using distance-based redundancy analysis, and statistical significance was determined using 9,999 Monte Carlo permutations. Potential interactions among taxa were revealed through co-occurrence network analysis, and correlations with Spearman’s |r| > 0.6 and *P* < 0.05 were considered statistically significant.

## Metabolomic analysis

For solid samples, 100 mg fresh material was placed into a 2-mL centrifuge tube containing a 6-mm steel bead, followed by the addition of 800 µL extraction solution (methanol: water = 4:1, v/v) supplemented with an internal standard (L-2-chlorophenylalanine, 0.02 mg/mL). The samples were ground using a tissue grinder at − 10 °C and 50 Hz for 6 min and subjected to low-temperature ultrasonic extraction at 5 °C and 40 kHz for 30 min. The extracts were incubated at − 20 °C for 30 min and centrifuged at 13,000 × *g* for 15 min at 4 °C, after which the supernatant was transferred into autosampler vials for liquid chromatography–tandem mass spectrometric analysis. For liquid samples, 100 µL was mixed with 400 µL extraction solution (acetonitrile: methanol = 1:1, v/v) containing the internal standard, vortexed for 30 s, ultrasonically extracted for 30 min at 5 °C, and then treated as described above. The resulting extracts were dried under nitrogen gas and reconstituted in 100 µL acetonitrile: water (1:1, v/v) prior to injection. Quality control (QC) samples were prepared by pooling equal aliquots from each sample and injecting them at regular intervals (one QC sample for every 5–10 samples) to evaluate the stability and repeatability of the analytical process. Metabolomic profiling was conducted on a UHPLC–Orbitrap Exploris 240 mass spectrometer (Thermo Fisher Scientific, San Jose, CA, USA) equipped with an HSS T3 column (100 × 2.1 mm, 1.8 μm; Waters, Milford, MA, USA). The mobile phases comprised phase A (water: acetonitrile = 95:5 v/v, containing 0.1% formic acid) and phase B (acetonitrile: isopropanol: water = 47.5:47.5:5 v/v/v, containing 0.1% formic acid). The flow rate, column temperature, and the injection volume were set to 0.4 mL/min, 40 °C, and 3 µL, respectively. The mass spectrometer was operated in both positive and negative ion modes with a scan range of m/z 70–1050. The key source parameters included a spray voltage of + 3500 V/–3000 V, sheath gas flow of 50 arb, auxiliary gas flow of 13 arb, capillary temperature of 450 °C, and stepped collision energies of 20/40/60 V. Raw data were processed using Progenesis QI (Waters, Milford, USA) for baseline filtering, peak detection, integration, correction of retention time, and peak alignment.

Differential metabolites were identified using multivariate and univariate analyses. Principal component analysis (PCA) and orthogonal partial least squares discriminant analysis (OPLS-DA) were performed using the R package ropls v.1.6.2 to visualize the differences in metabolic profiles among treatments. The reliability of the OPLS-DA model was evaluated using seven-fold cross-validation and permutation tests. Variables with a variable importance in projection (VIP) score > 1.0 from the OPLS-DA model, and *P* < 0.05, determined using Student’s *t*-tests, were considered statistically significant differential metabolites. Pathway enrichment analysis was conducted using the Kyoto Encyclopedia of Genes and Genomes (KEGG, https://www.kegg.jp/kegg/pathway.html) database. Differential metabolites were mapped to KEGG pathways, and enrichment significance was assessed using Fisher’s exact test, with adjusted *P* < 0.05 considered statistically significant. The results were visualized using bubble plots and enrichment maps to highlight the key metabolic pathways affected by different treatments.

### Statistical analysis

Statistical analyses were performed using IBM SPSS Statistics v.22 (IBM Corp., Armonk, NY, USA). Differences among treatments were assessed by one-way analysis of variance under the General Linear Model procedure. The applied model was:

1 $$Y =\mu\;+\;T_i\;+\;\varepsilon_{ij},$$

where Y represents the observed response variable; µ is the overall mean; T_i_ denotes the fixed effect of treatment; and ε_ij_ is the residual error term. Duncan’s multiple range test was used for post-hoc comparisons, and differences were considered significant at *P* < 0.05.

For the microbial community structure and metabolomics, PCoA based on Bray–Curtis distances was used to visualize the variation among treatments. PERMANOVA was performed to evaluate the statistical significance of treatment separation. LEfSe was used to identify taxa with differential abundances (LDA score > 2.0, *P* < 0.05). For metabolomic data, multivariate analyses, including PCA and OPLS-DA, were performed, and differential metabolites were identified with VIP > 1.0 and *t*-test (*P* < 0.05). Pathway enrichment analysis was conducted against the KEGG database using Fisher’s exact test. All data were visualized using OriginPro v.2021b (OriginLab Corp., Northampton, MA, USA).

## Results

### Disease significantly altered the fermentability of raw materials and characteristics of initial microbial communities

Before ensiling, plants infected with leaf blight exhibited nutritional and microbial changes that were unfavorable for feeding value and silage fermentation quality (Table 1). Compared with CON, DCON had significantly lower crude protein content (7.06% vs. 8.91% DM, *P* = 0.001) and higher NDF and ADF contents (52.5% and 32.1% DM, respectively in DCON vs. 46.1% and 28.2% DM in CON, *P* < 0.05). In addition, the WSC content markedly decreased by leaf blight (88.7 vs. 119 g/kg DM, *P* = 0.016). Accompanying these nutritional shifts, LAB abundance was significantly lower (3.22 vs. 4.22 log10 CFU/g FM, *P* = 0.022), whereas mold counts sharply increased (4.25 vs. 3.22 log10 CFU/g FM, *P* = 0.001) in DCON than in CON. These findings indicated that diseased maize was characterized by insufficient fermentable substrates.

**Table 1:** Nutritional value and epiphytic microbes in healthy vs. leaf blight-infected maize

Items	Healthy	Leaf blight	Standard error of the means	*P*
Dry matter (%, FM)	31.7 ± 0.23	35.2 ± 0.37	0.81	0.334
Crude protein (% DM)	8.91 ± 0.53a	7.06 ± 0.24b	0.49	0.001
Neutral detergent fiber (% DM)	46.1 ± 0.72b	52.5 ± 0.50a	1.47	0.033
Acid detergent fiber (% DM)	28.2 ± 0.25b	32.1 ± 0.94a	0.94	0.002
Water-soluble carbohydrates (% g/kg DM)	119 ± 8.17a	88.7 ± 0.64b	7.71	0.016
pH	6.28 ± 0.06b	6.36 ± 0.04a	0.04	0.021
Buffering capacity (mE/kg DM)	29.4 ± 3.10	21.4 ± 1.35	2.36	0.080
Aerobic bacteria (log10 CFU/g FM)	7.26 ± 0.03	7.05 ± 0.18	0.09	0.309
Lactic acid bacteria (log10 CFU/g FM)	4.22 ± 0.06a	3.22 ± 0.27b	0.26	0.022
Yeasts (log10 CFU/g FM)	3.27 ± 0.10	3.33 ± 0.11	0.11	0.707
Molds (log10 CFU/g FM)	3.22 ± 0.13b	4.25 ± 0.04a	0.24	0.001

Different lowercase letters in the same raw represent significant difference between treatments (*P* < 0.05)

## Disease suppressed lactic acid fermentation and promoted proteolysis, whereas LAB inoculation restored fermentation balance

The DCON showed elevated pH, significantly reduced lactic acid content, and markedly increased butyric acid content compared with those of the CON (*P* < 0.05), indicating that lactic acid fermentation was suppressed, and undesirable butyric acid fermentation occurred. In contrast, inoculation with *L. plantarum* (HT1) led to the lowest pH, highest lactic acid content, and significant inhibition of butyric acid production, resulting in an overall fermentation quality superior to those of CON and DCON (Table 2).

**Table 2:** Effects of different treatments on the fermentation quality of whole-plant maize silage

Treatment	pH	Microbial quantity (log10 CFU/g FM)				Organic acids (g/kg DM)			
		Aerobic bacteria	Lactic acid bacteria	Yeasts	Molds	Lactic acid	Acetic acid	Propionic acid	Butyric acid
CON	4.28 ± 0.16b	6.22 ± 0.02a	6.40 ± 0.01b	2.50 ± 0.01b	3.64 ± 0.02b	20.5 ± 1.36b	2.35 ± 1.19	0.91 ± 0.04	0.46 ± 0.06b
DCON	4.64 ± 0.08a	6.17 ± 0.01b	6.29 ± 0.01c	2.54 ± 0.01a	3.54 ± 0.03c	4.91 ± 2.42c	1.46 ± 0.91	0.16 ± 0.01	0.81 ± 0.10a
HT1	4.01 ± 0.29c	6.18 ± 0.01ab	7.11 ± 0.01a	2.50 ± 0.003b	3.79 ± 0.04a	35.1 ± 6.03a	5.41 ± 0.68	0.98 ± 0.23	0.11 ± 0.03c
Standard error of the means	0.13	0.01	0.13	0.01	0.04	4.76	0.76	0.22	0.11
P	0.005	0.049	< 0.0001	0.004	0.003	0.004	0.057	0.646	0.001

Different lowercase letters in the same column represent significant difference between treatments (*P* < 0.05). CON, healthy maize silage; DCON, maize silage infected with leaf blight; HT1, maize silage infected with leaf blight and supplemented with *L. plantarum*

The nitrogen fractions further revealed that disease accelerated protein hydrolysis (Table 3). DCON had the highest NH_3_–N content (17.2 g/kg TN), exceeding those of CON (12.3 g/kg TN) and HT1 (10.3 g/kg TN, *P* = 0.005), with FAA-N levels following a similar trend. Correspondingly, protease activities were significantly enhanced in the DCON. Aminopeptidase and carboxypeptidase activities increased to 46.4 and 167 U·h^− 1^·g FM^− 1^, respectively, which were much higher than those of the CON (27.4 and 140 U·h^− 1^·g FM^− 1^). Acid, cysteine, and aspartic protease activities were also significantly elevated in the DCON (*P* < 0.05). However, all these enzyme activities were significantly lower in the HT1 than in the DCON, and most were close to or even lower than those in the CON.

**Table 3:** Effects of different treatments on nitrogenous compounds in silage

Treatment	Free amino acid nitrogen (g/kg FM)	Ammonia-*N* (g/kg TN)	Free amino acid (g/kg FM)	Protease activities (units/h/g/FM)					
				Aminopeptidase	Carboxypeptidase	Acid protease	Cysteine protease	Aspartate protease	Serine protease
CON	101 ± 14.6	12.3 ± 0.24b	231 ± 5.85b	27.4 ± 1.09b	140 ± 3.76b	82.2 ± 1.93	6.68 ± 0.31b	5.20 ± 0.03b	2.34 ± 0.21b
DCON	76.6 ± 4.75	17.2 ± 1.63a	330 ± 10.9a	46.4 ± 1.18a	167 ± 4.13a	85.1 ± 0.94	8.06 ± 0.41a	6.10 ± 0.17a	3.53 ± 0.29a
HT1	61.8 ± 9.09	10.3 ± 0.19b	208 ± 10.1b	21.4 ± 1.93c	99.7 ± 1.70c	75.0 ± 4.37	5.50 ± 0.45b	4.56 ± 0.36b	2.15 ± 0.23b
Standard error of the means	7.75	1.14	19.3	3.84	9.99	2.05	0.42	0.36	0.25
P	0.096	0.005	< 0.0001	< 0.0001	< 0.0001	0.104	0.011	0.009	0.015

Different lowercase letters in the same column represent significant difference between treatments (*P* < 0.05). CON, healthy maize silage; DCON, maize silage infected with leaf blight; HT1, maize silage infected with leaf blight and supplemented with *L. plantarum*

### LAB inoculation reshaped silage bacterial community structure and stability under leaf blight, decoupling proteolysis from community dynamics

**Fig. 1:**
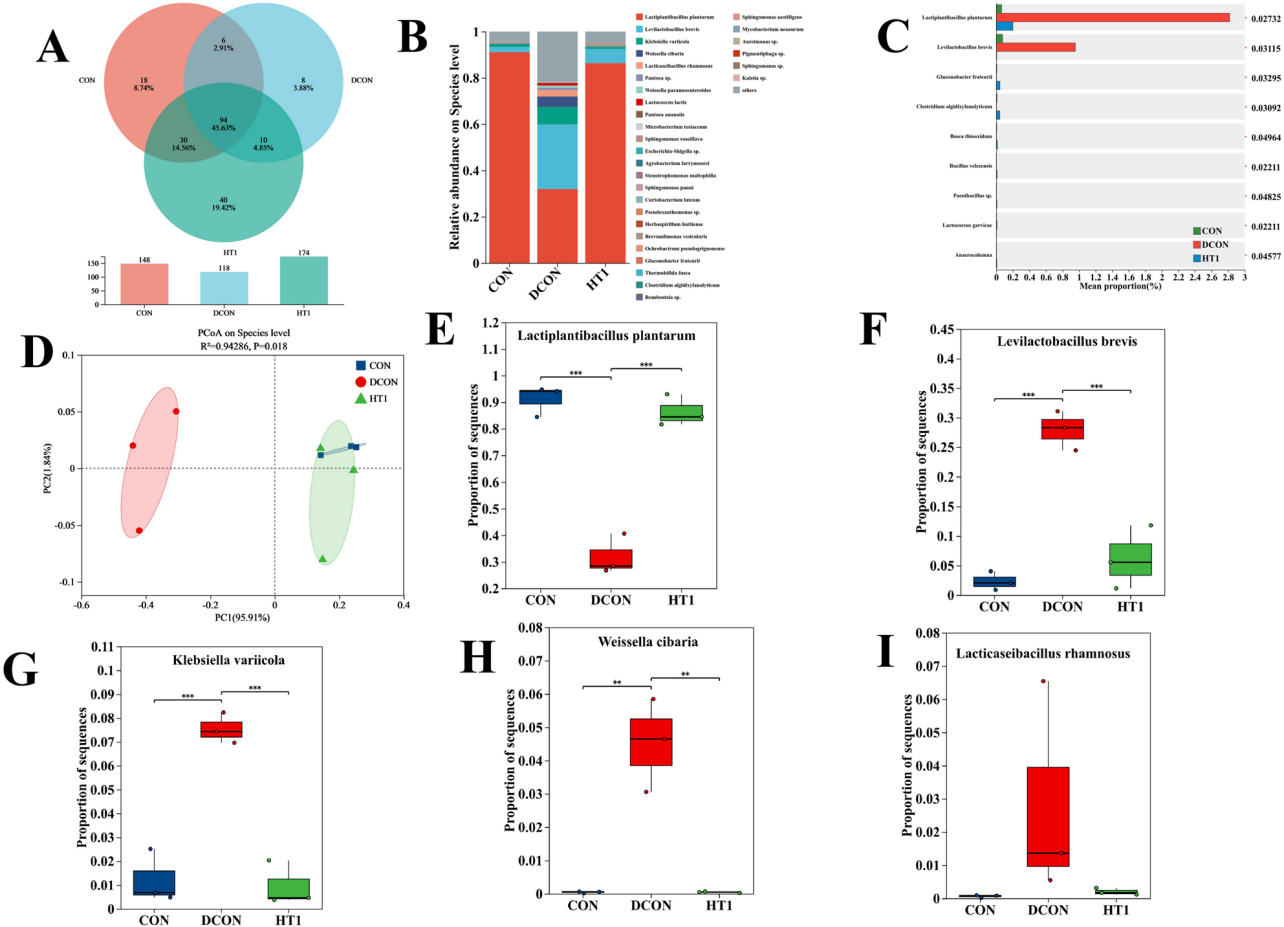
Effects of different treatments on bacterial diversity and relative abundance. Note: **A**, different colors represent different treatments; **B**, the X-axis represents different treatments, and the Y-axis represents bacterial relative abundance; **C**, the X-axis represents bacterial relative abundance, and the Y-axis represents bacterial taxa; **D**, ellipses indicate the 95% confidence interval; **E**-**I**, the X-axis represents treatments, and the Y-axis represents bacterial relative abundance. ** and *** indicate *P* < 0.01 and *P* < 0.001, respectively. CON, healthy maize silage; DCON, maize silage infected with leaf blight; HT1, maize silage infected with leaf blight and supplemented with *L. plantarum*

**Fig. 2:**
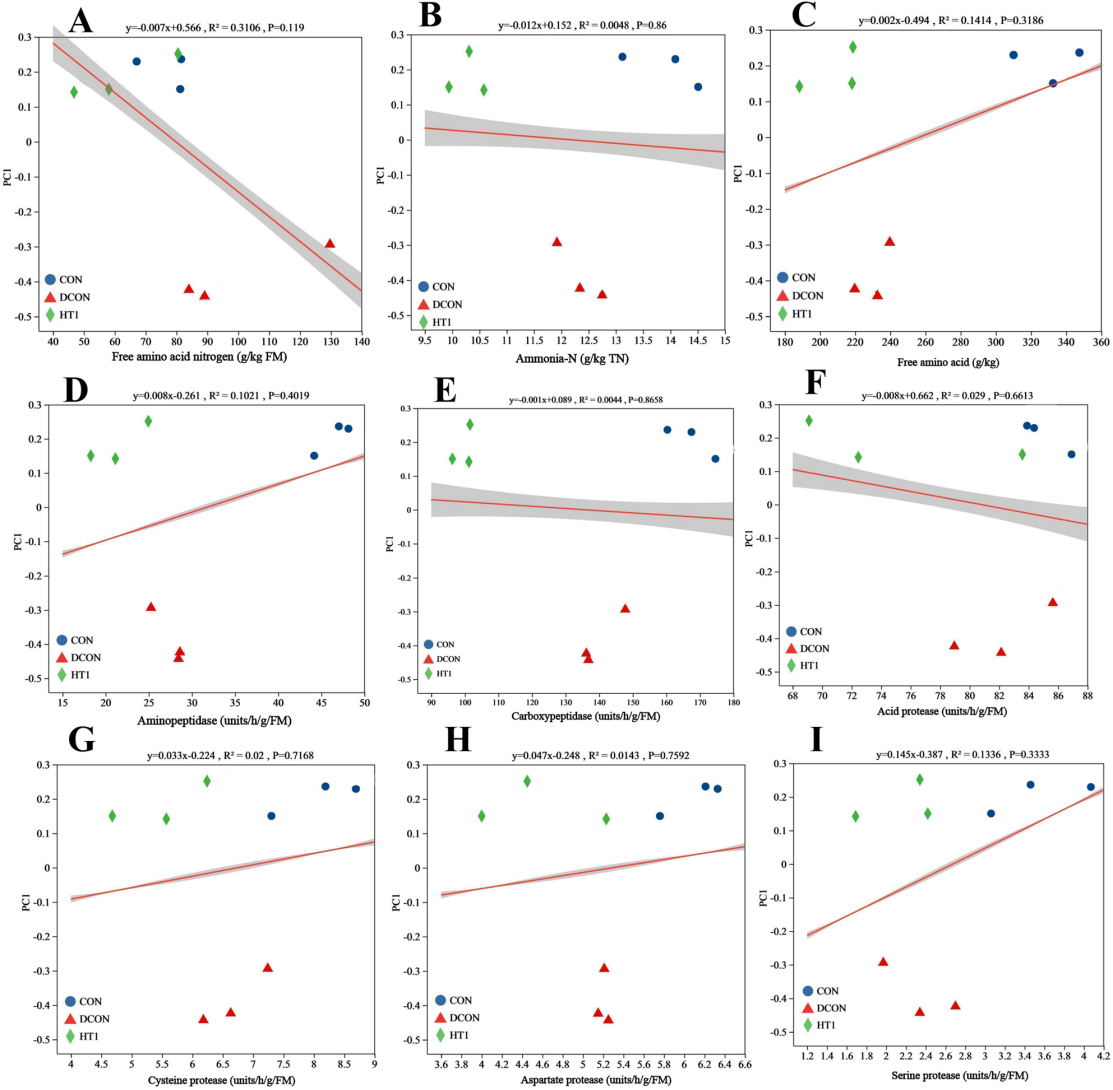
Ordination regression analysis between environmental factors and bacterial beta diversity. Note: the X-axis represents environmental factors, and the Y-axis represents the beta diversity ordination axis. *R*^2^ indicates the coefficient of determination. Projections represent the 95% confidence interval

Different treatments altered the bacterial community structure and composition of the silage (Fig. 1). At the OTU level (Fig. 1A), 148, 118, and 174 OTUs were detected in the CON, DCON, and HT1 treatments, respectively, with 94 OTUs (45.63%) shared among the three treatments. *L. plantarum*, *L. brevis*, *Klebsiella*, *Weissella*, and *L. rhamnosus* were the five most abundant taxa (Fig. 1B). The relative abundances of the nine major bacterial species significantly differed among the treatments (*P* < 0.05; Fig. 1C). The bacterial community in the DCON treatment was completely separated from those in the CON and HT1 treatments within the 95% confidence interval (*R*^2^ = 0.943, *P* = 0.018; Fig. 1D). Specifically, the relative abundance of *L. plantarum* was significantly lower (Fig. 1E), whereas the abundances of *L. brevis*, *K. variicola*, *W. cibaria*, and *L. rhamnosus* were significantly higher (Fig. 1F–I) in the DCON treatment than in the CON and HT1 treatments. Based on ordination regression analysis, FAA-N exerted the highest explanatory power on community diversity (*R*^2^ = 0.3106, *P* = 0.119; Fig. 2).

**Fig. 3:**
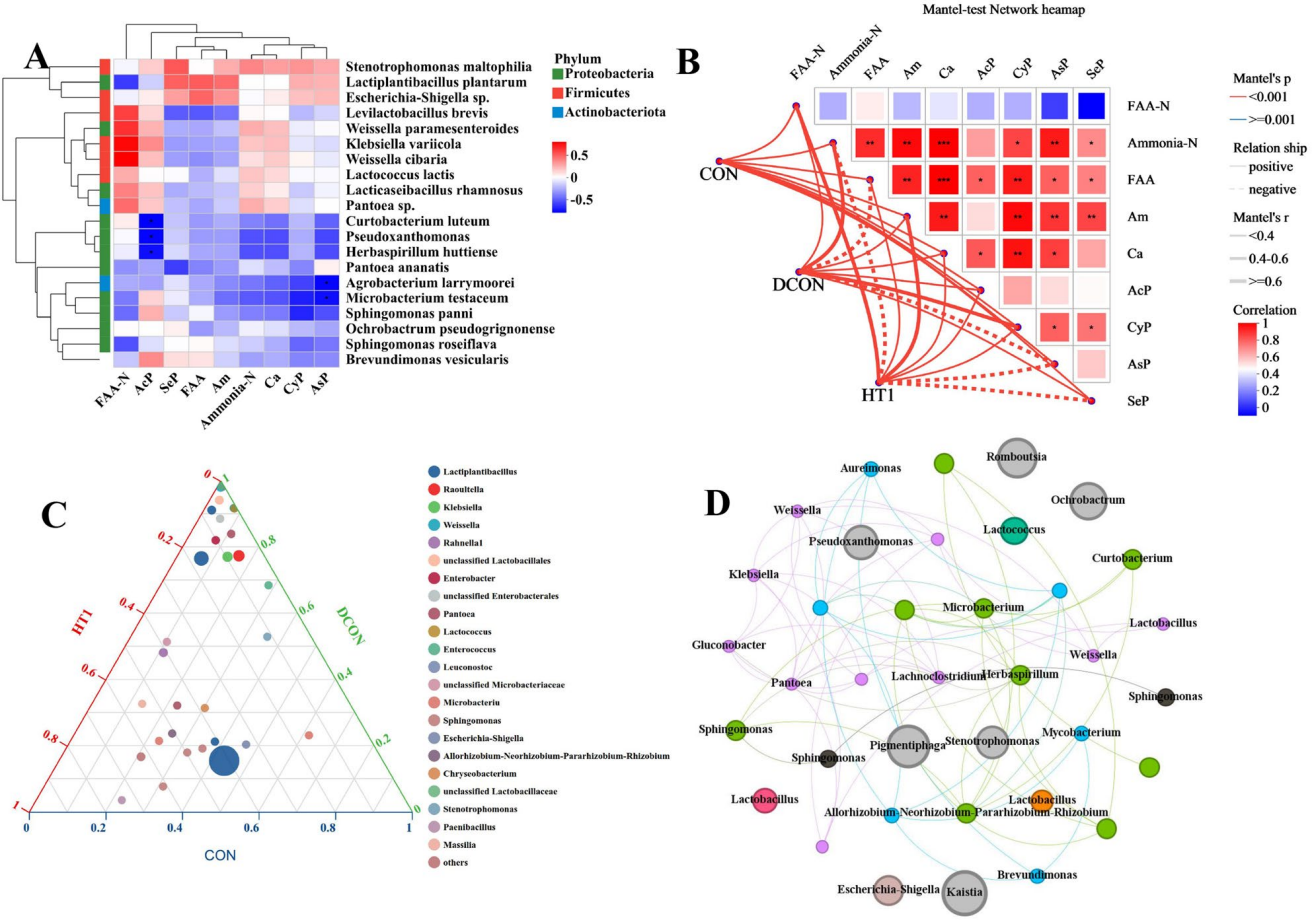
Heatmap (**A**) of bacterial communities and environmental factors, mantel-test network (**B**), ternary plot (**C**), and co-occurrence network (**D**) of bacterial communities. Note: the X-axis represents environmental factors, and the Y-axis represents bacterial taxa. **B**, *, **, and *** indicate *P* < 0.05, 0.01, and 0.001, respectively. **C**, solid circles in the figure represent species at the genus level, and the size of the circles represents the average relative abundance of each species. **D**, the size of the nodes represents species abundance, and different colors indicate different species. CON, healthy maize silage; DCON, maize silage infected with leaf blight; HT1, maize silage infected with leaf blight and supplemented with *L. plantarum*

In the correlation analysis between amino acid metabolites and protein degradation indices (Fig. 3A), the relative abundances of *C. luteum* and *H. huttiense* were significantly negatively correlated with acid protease activity, whereas *A. larrymoorei* and *M. testaceum* were negatively correlated with aspartate protease activity. Mantel-test network analysis (Fig. 3B) revealed that nitrogenous compounds and protease-associated bacteria were significantly coupled with the bacterial community structure (*P* < 0.001). Notably, inoculation with *L. plantarum* (HT1) weakened the coupling strength between the bacterial communities and proteolysis. The ternary plot (Fig. 3C) demonstrated pronounced differences in community composition: CON was enriched with LAB (e.g., *Lactiplantibacillus*) and some beneficial genera; DCON harbored relatively high abundances of potential proteolytic genera such as *Escherichia-Shigella* and *Pantoea*; and HT1 shifted the community structure toward the healthy level by reducing the abundance of pathogenic genera. Co-occurrence network analysis (Fig. 3D) further demonstrated that *Lactiplantibacillus* occupied the network core and formed positive associations with *Weissella*, *Lactococcus*, and *Sphingomonas*. By contrast, the associations of *Escherichia–Shigella* and *Klebsiella* were strengthened in the DCON treatment, suggesting their involvement in abnormal nitrogen metabolism and proteolysis. The HT1 treatment showed increased overall complexity of microbial interactions.

**Fig. 4:**
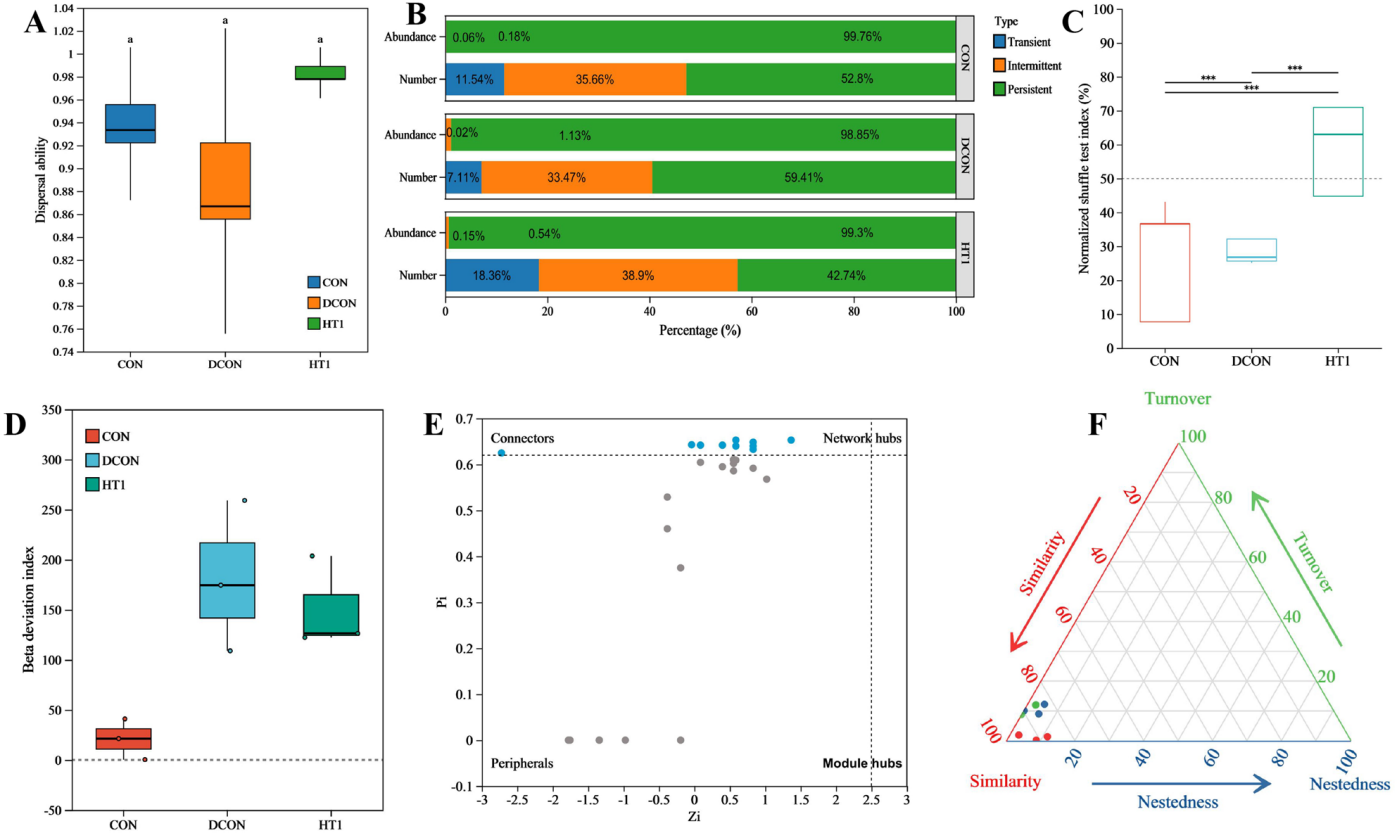
Effects of different treatments on dispersal ability (**A**), core community identification (**B**), normalized shuffle test index (**C**), beta deviation index (**D**), Zi–Pi of species interactions (**E**), and beta diversity patterns (**F**). Note: A, the X-axis represents treatments, and the Y-axis represents dispersal ability values. B, different colors indicate the average relative abundance proportion of three types of species: transient, intermediate, or persistent. C, the Y-axis represents the normalized shuffle test index. D, the X-axis represents treatments, and the Y-axis represents the beta deviation index. E, nodes in the figure represent species. Based on topological features, nodes are classified into four types: Module hubs (highly connected within modules, Zi > 2.5 and Pi < 0.62), connectors (highly connected between modules, Zi < 2.5 and Pi > 0.62), network hubs (highly connected in the entire network, Zi > 2.5 and Pi > 0.62), and peripherals (low connectivity both within and between modules, Zi < 2.5 and Pi < 0.62). Nodes of the first three types are considered key nodes. Nodes are colored according to type: blue for connectors and black for Peripherals. F, contributions of beta diversity components-similarity, turnover, and nestedness—to community ecological processes. Each point represents a pair of samples, and its position is determined by the mean values of similarity, species turnover, and nestedness matrices, with each ternary component summing to 1. CON, healthy maize silage; DCON, maize silage infected with leaf blight; HT1, maize silage infected with leaf blight and supplemented with *L. plantarum*

Community stability and core microbiota analyses (Fig. 4) indicated that the dispersal ability of the bacterial communities in the CON and HT1 treatments was higher than that in the DCON treatment (Fig. 4A). Core microbiota analysis (Fig. 4B) revealed that persistent species dominated across treatments; however, the proportions of transient and intermediate species were reduced in the DCON treatment. The normalized shuffle test index (Fig. 4C) was significantly higher in the HT1 treatment than in the CON and DCON treatments (*P* < 0.001). The β-deviation index (Fig. 4D) fluctuated most in the DCON treatment, whereas that of the HT1 treatment was intermediate between those of the CON and DCON treatments. Species interaction topology (Fig. 4E) indicated that most nodes in the DCON were peripheral species, whereas the HT1 had relatively high number of connector nodes, increasing the potential for cross-module interactions. β-diversity partitioning (Fig. 4F) further demonstrated that community differences among the three treatments were mainly driven by species turnover; however, the community structure of the HT1 was closer to that of the CON, indicating its positive role in reducing community replacement rates and maintaining structural stability.

### Changes in metabolite abundance and differences in proteolysis-related pathways under different treatments

**Fig. 5:**
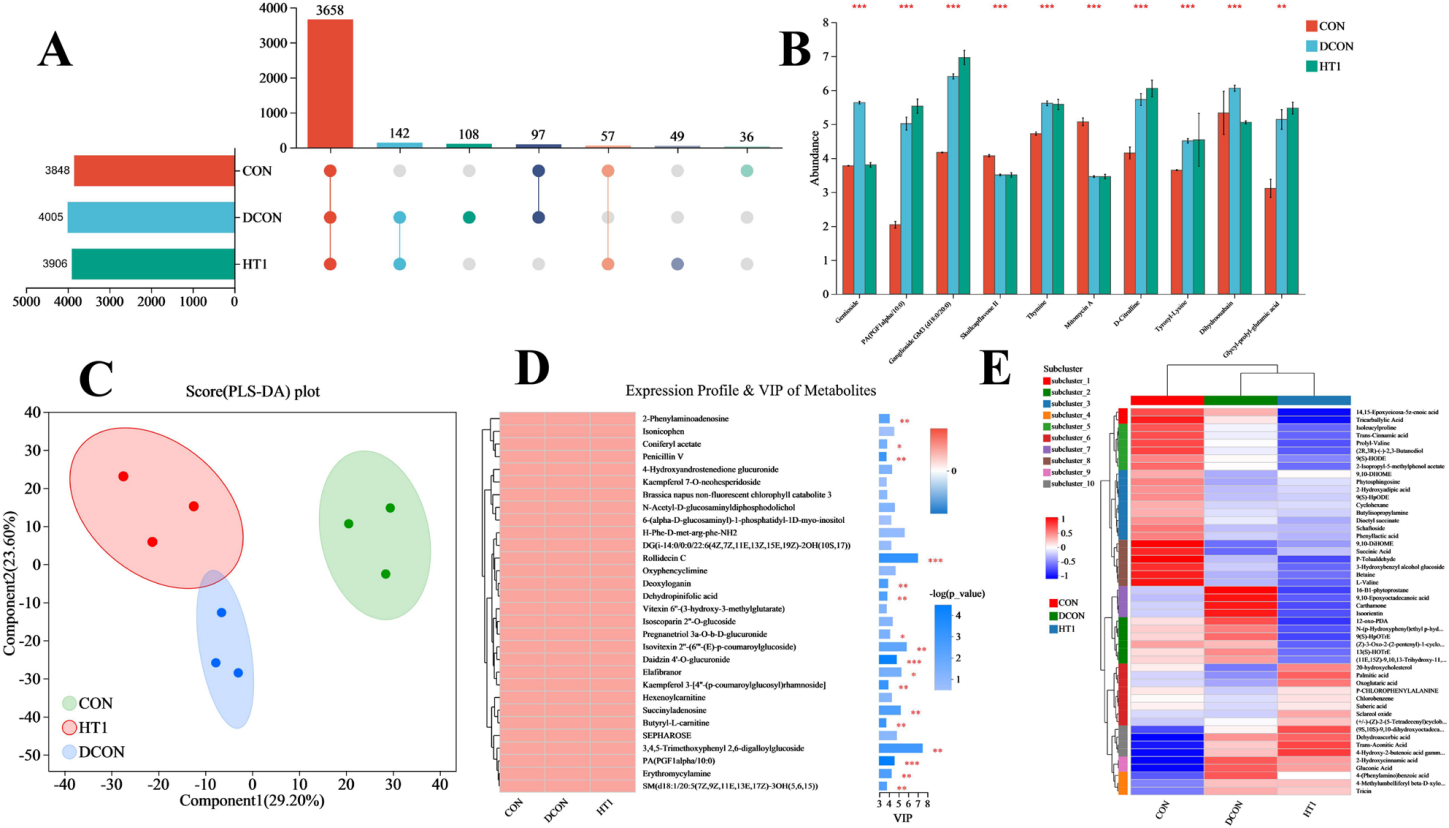
Effects of different treatments on metabolite number and differences. Note: **A**, Different colors represent different treatments. **B**, the Y-axis represents metabolite names, and the X-axis represents the average relative abundance of metabolites in different groups; the far right shows *P*-values, with ** indicating 0.001 < *P* ≤ 0.01 and *** indicating *P* ≤ 0.001. **C**, PLS-DA score plot. Greater separation between the two groups of samples indicates a more significant classification effect. **D**, *, **, and *** indicate *P* < 0.05, 0.01, and 0.001, respectively. **E**, the dendrogram on the left represents clustering of metabolites, with metabolite names on the right; the dendrogram on the top represents sample clustering, with sample names at the bottom. CON, healthy maize silage; DCON, maize silage infected with leaf blight; HT1, maize silage infected with leaf blight and supplemented with *L. plantarum*

In total, 3,348, 4,205, and 3,508 metabolites were detected in the CON, DCON, and HT1 treatments, respectively, with DCON exhibiting the highest number of metabolites (Fig. 5A). Numerous shared and certain unique metabolites were noticed among the treatment treatments. The DCON treatment showed significantly higher levels of several metabolites than did the CON treatment, particularly those associated with amino acid metabolism and protein degradation (Fig. 5B). In contrast, the HT1 treatment exhibited a decreasing trend for most metabolites, with levels closer to those of the CON treatment. The PLS-DA plot revealed clear separation of metabolic profiles among the three treatments, with the highest divergence observed between the DCON and CON treatments (Fig. 5C). VIP analysis of differential metabolites (Fig. 5D) indicated that multiple amino acid metabolites and organic acids strongly contributed to discrimination among treatments. Specifically, metabolites related to proteolysis, such as glutamic acid, aspartic acid, and their derivatives, were significantly enriched in the DCON treatment, whereas their abundance was markedly reduced in the HT1 treatment. Hierarchical clustering analysis (Fig. 5E) further demonstrated that metabolite expression pattern of the DCON treatment most markedly differed from that of the CON treatment, whereas metabolite profile of the HT1 treatment was relatively similar to that of the CON treatment.

**Fig. 6:**
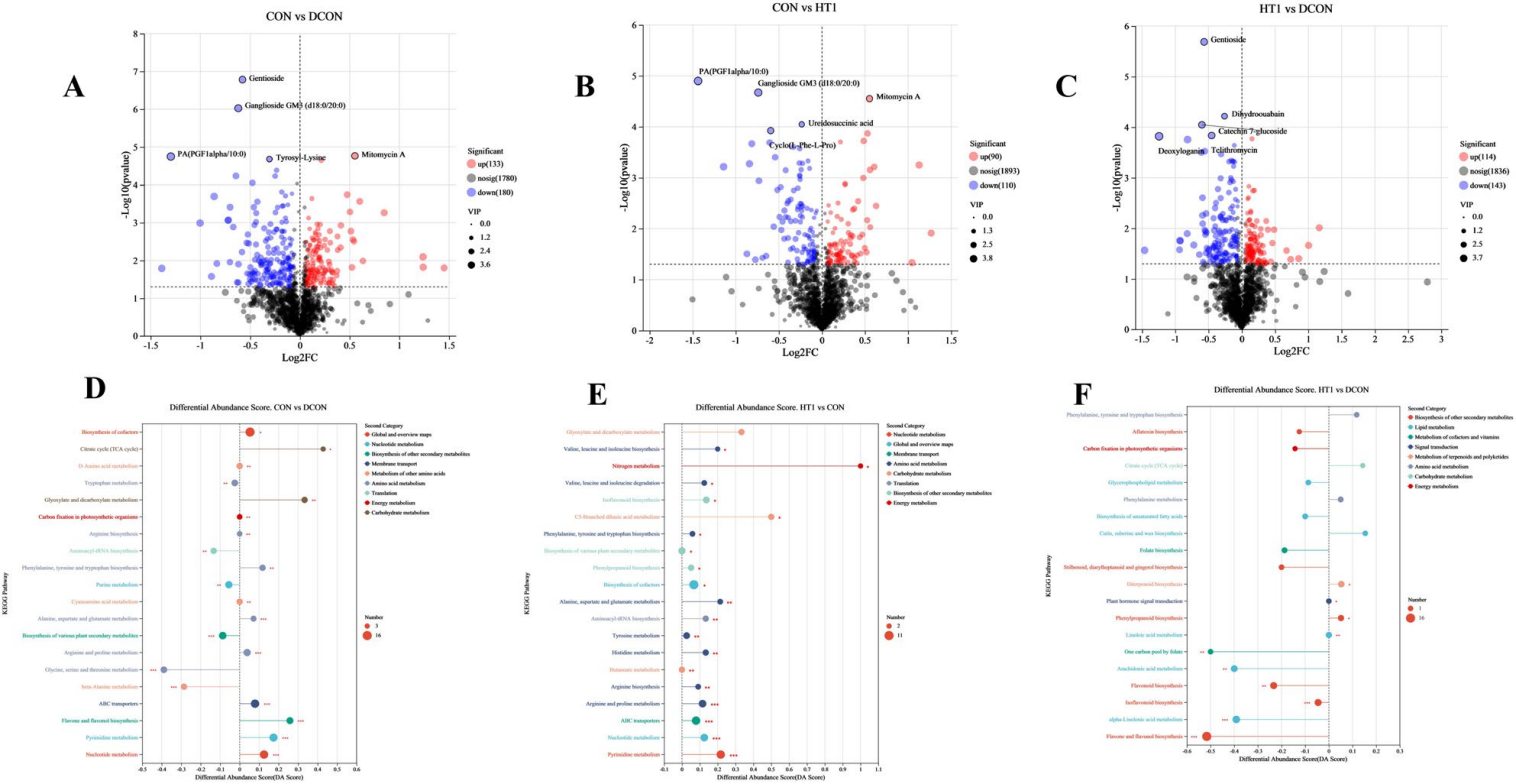
Effects of different treatments on metabolites and KEGG pathway enrichment. Note: **A**-**C**, the X-axis represents the fold change of metabolite expression between two groups, and the Y-axis represents the statistical significance of differences in metabolite expression. Each point represents a specific metabolite, and the size of the point indicates its VIP value. **D**-**F**, the X-axis represents the differential abundance score (DA Score), and the Y-axis represents the names of KEGG metabolic pathways. The DA Score reflects the overall change of all metabolites in a pathway, with a score of 1 indicating that all annotated differential metabolites in the pathway are upregulated, and − 1 indicating that all are downregulated. The length of the line represents the absolute value of the DA Score. The size of the circle represents the number of annotated differential metabolites in the pathway, with larger circles indicating more differential metabolites. Circles located to the right of the central axis with longer lines indicate a pathway overall tending toward upregulation, whereas circles to the left with longer lines indicate a pathway overall tending toward downregulation. CON, healthy maize silage; DCON, maize silage infected with leaf blight; HT1, maize silage infected with leaf blight and supplemented with *L. plantarum*

Different treatments significantly shaped the metabolite composition and pathway enrichment patterns of maize silage (Fig. 6). At the metabolite level, many compounds were upregulated in the DCON treatment (Fig. 6A), including several compounds closely associated with amino acid metabolism and proteolysis, indicating that the disease markedly accelerated protein breakdown and disrupted nitrogen metabolic homeostasis. The metabolic profile of the HT1 treatment was relatively similar to that of the CON treatment (Fig. 6B), with most metabolites exhibiting comparable abundances, suggesting that inoculation with *L. plantarum* partially restored the metabolic characteristics of healthy materials. In contrast to the DCON treatment (Fig. 6C), the HT1 treatment displayed a clear reduction in proteolysis-related metabolites and increases in metabolites involved in organic acid and lipid metabolism, implying that inoculation counteracted disease-induced metabolic disturbances and optimized fermentation through pathway reprogramming. KEGG pathway enrichment analysis further revealed the functional implications of these changes. Under DCON conditions (Fig. 6D), the pathways involved in amino acid biosynthesis and degradation, such as valine, leucine, and isoleucine degradation, and glutamate metabolism, were overall upregulated, and those related to carbon fixation and energy metabolism fluctuated, reflecting disease-driven intensification of proteolysis and disruption of metabolic balance. In contrast, the differences between the HT1 and CON treatments were restricted to a small number of amino acid and nucleotide metabolic pathways (Fig. 6E), indicating that inoculation progressively restored the metabolic state toward a healthy level. Further comparison of the HT1 and DCON treatments (Fig. 6F) revealed that proteolysis-related pathways, including glutamate and aspartate metabolism, were significantly downregulated, whereas those associated with organic acid biosynthesis, lipid metabolism, and certain secondary metabolites were strongly upregulated. These results suggested that *L. plantarum* effectively alleviated disease-induced nitrogen metabolic disorders through metabolic network reprogramming and promoted pathways favorable for lactic acid fermentation.

## Discussion

### Foliar disease-induced nutritional and microbial alterations compromise the ensiling suitability of maize

This study revealed that leaf blight infection significantly affected the nutritional composition and microbial community of maize prior to ensiling, changes that were unfavorable for successful fermentation. Diseased materials were characterized by reduced crude protein content, low levels of WSC, elevated fiber fractions, and a concomitant decrease in LAB with a rapid expansion of molds. Collectively, these traits indicated that diseased plants entered the ensiling process with insufficient fermentable substrates, a disadvantageous initial LAB population, and an excessive fungal burden, thereby predisposing silage to a subsequent fermentation imbalance and aggravated proteolysis.

For instance, the decline in crude protein content was similar to that of a previous finding reporting that foliar diseases promote nitrogen remobilization and protein degradation [42], reducing the feeding value of silage materials. This effect is generally associated with pathogen-induced activation of proteases and impaired nitrogen assimilation in diseased tissues [43, 44]. Simultaneously, the increases in neutral and acid detergent fibers corroborate the findings of previous studies reporting that fungal infection elevates lignocellulosic fractions, thereby reducing forage health [45, 46]. The underlying mechanism may involve pathogen-induced cell wall fortification and lignin deposition, which serve as host defense responses but also reduce the availability of soluble carbohydrates to ruminal microbes [47, 48]. The pronounced decline in WSC observed in the present study was particularly critical for fermentation quality. WSC are the principal substrates for rapid lactic acid production by LAB and for lowering pH, and their depletion directly limits fermentation efficiency [12, 21]. Similar reductions in WSC have been reported under pathogen infection, in which carbon allocation is redirected from storage metabolism toward defense-related pathways, thereby decreasing the availability of sugars for fermentation [49, 50]. Such a metabolic shift diminishes lactic acid synthesis and weakens the competitive advantage of LAB against undesirable microbes, ultimately increasing the risk of fermentation failure.

Alterations in microbial communities further highlighted the vulnerability of diseased materials. The reduced LAB population weakened the capacity of rapid acidification during the early ensiling stage, and mold proliferation increased the likelihood of quality deterioration. LAB play a crucial role in quickly lowering pH to inhibit spoilage organisms at the onset of ensiling [51]. However, when LAB are scarce, the environment becomes highly susceptible to colonization by clostridia and fungi, intensifying proteolysis and spoilage. The increased mold load was consistent with those of earlier reports that *Fusarium* [52, 53] and *Aspergillus* [54] often proliferate in damaged plant tissues prior to ensiling [55]. This is particularly concerning as research indicates that even after 60 days of storage, the potential for microbial contamination and pathogens remains a significant risk in high-moisture forages [56, 57]. These fungi compete with LAB for limited sugars and secrete proteases and mycotoxins, accelerating silage deterioration through nutritional depletion and biological toxicity.

In summary, leaf blight undermined the nutritional–microbial balance required for successful ensiling through a combined mechanism of substrate depletion, microbial imbalance, and enhanced protease activity. These findings corroborate those of previous reports emphasizing that the ensiling process is highly sensitive to the initial microbial ecology and substrate composition [58, 59]. Importantly, the present study provides direct evidence that foliar disease-induced alterations in nutrients and microbial load exert additive effects that substantially increase the risk of fermentation imbalance and excessive proteolysis. However, microbial analyses in this study were restricted to culturable treatments, which might have underestimated the roles of non-culturable or low-abundance taxa in proteolysis. Future studies integrating high-throughput sequencing of epiphytic microbiota will be essential for fully elucidating the molecular mechanisms underlying silage deterioration under disease stress and guiding the development of effective strategies for managing silage production from diseased crops.

### LAB inoculation reshapes fermentation trajectory and mitigates disease-induced proteolysis

In this study, relatively high pH, reduced lactic acid content, and marked accumulation of butyric acid in the DCON treatment indicated that the rapid acidification window during the early stage of ensiling was disrupted. LAB failed to establish an acidic barrier in time, thereby allowing an anaerobic but relatively high-pH environment to favor the activation of clostridial butyric fermentation pathways [60]. In contrast, the HT1 treatment exhibited a typical homolactic fermentation pattern, characterized by the lowest pH value, highest lactic acid concentration, and strong inhibition of butyric acid, suggesting that *L. plantarum* rapidly colonized and efficiently utilized substrates to reshape the fermentation trajectory toward the desired “lactic acid–dominated rapid pH decline” pathway, which is consistent with those of previous reports [61, 62]. Nitrogen fractions and protease activities further substantiated this process. In the DCON treatment, the accumulation of NH_3_–N and FAA-N was accompanied by increases in the activities of aminopeptidase, carboxypeptidase, and several endopeptidases, indicating intensified proteolysis. This could be attributed to pathogen-induced release of host proteases and expansion of proteolytic bacterial groups [63]. Moreover, delayed acidification prolonged the period, in which pH remained within the optimal range for enzymatic activity, resulting in a positive feedback loop between fermentation and proteolysis. Conversely, significantly reduced protease activities and nitrogen losses in the HT1 treatment were consistent with the cascade effect of “acidification–enzyme suppression–restricted nitrogen loss.” This was attributed to the direct inhibition of microbial protease activity under acid stress and competitive dominance of *L. plantarum*, which suppressed the expansion of proteolytic bacteria [64, 65].

The bacterial community structure provided further mechanistic insights. In the DCON treatment, *L. plantarum* abundance was markedly suppressed, whereas the abundances of subdominant taxa, such as *L. brevis*, *K. variicola*, *W. cibaria*, and *L. rhamnosus*, increased, along with the enrichment of potential proteolytic genera such as *Escherichia–Shigella* and *Pantoea*. These community shifts aligned with the functional phenotypes of butyric acid accumulation and enhanced proteolysis [66]. Notably, *Klebsiella* species remain metabolically active under low-acid conditions and may harbor nitrogen assimilation and regulation networks, thereby exacerbating nitrogen imbalance in sub-acidic environments [67–69]. Co-occurrence network analysis further revealed that the DCON communities were dominated by peripheral nodes with weakened cross-module connectivity, indicating reduced ecological stability. In contrast, in the HT1 treatment, reestablishment of *Lactiplantibacillus* as a central node formed strong positive associations with *Weissella* and *Lactococcus* and increased the connector nodes, which enhanced community connectivity and robustness. These findings suggest that the effect of *L. plantarum* extends beyond acid production, manifesting in community network restructuring that decouples the association between proteolytic bacteria and nitrogen metabolic disorders, thereby stabilizing the fermentation trajectory at the system level [70–72].

Substrate availability is another critical factor in fermentation. The disease significantly reduced WSC and increased the lignocellulosic fractions, which impaired the efficiency of lactic acid fermentation, prolonged the time required for acidification, and created a temporal window for butyric acid fermentation. The advantage of HT1 lay in its ability, even under limited substrate conditions, to exploit high substrate affinity and homolactic efficiency to rapidly accumulate lactic acid, lowering the pH below the inhibitory threshold and blocking the cascade of “high pH–protease activation–expansion of proteolytic bacteria” [73, 74]. Moreover, LAB inoculation can reprogram carbon flow, reduce amino acid degradation, and enhance organic acid and energy metabolic pathways, thereby reducing the metabolic drivers of proteolysis at the network level [75, 76]. These findings align well with the reduced NH_3_–N and FAA-N levels and protease activities observed in the present study. Notably, microbial analyses in this study were mainly based on culturable techniques and relative abundance data, which might have underestimated the role of low-abundance but ecologically critical taxa. Furthermore, because the combined effects of disease and environmental factors may alter substrate thresholds and clostridial sensitivity, practical silage management should adopt integrated strategies, such as high inoculation rates of homolactic strains combined with optimized early DM management, to shorten the acidification constant and suppress the proteolytic window [77].

### Metabolic reprogramming by *L. plantarum* mitigates disease-induced proteolysis in maize silage

This study revealed the profound impact of leaf blight disease on the fermentation quality of corn silage and its nitrogen metabolic network. The DCON treatment showed a significant enrichment in amino acids and their derivatives (such as glutamate and aspartate), reflecting a strongly enhanced protein degradation process and the disruption of nitrogen metabolic homeostasis. This phenomenon can be attributed to tissue damage and increased buffer capacity caused by the disease, which prolong a high-pH state during the early stage of ensiling, triggering the cascade: high pH–protease activation–intensified amino acid degradation [78]. KEGG enrichment further confirmed significant upregulation of pathways, such as valine, leucine, and isoleucine degradation and glutamate metabolism [79], which represents an imbalance in nitrogen metabolism and suggests passive activation of unfavorable anaerobic metabolic pathways that promote the accumulation of NH_3_–N and generation of biogenic amines [55, 80]. The data on nitrogen components and protease activities were consistent with the metabolomic results, indicating that the disease amplified the driving force for proteolysis and magnified nitrogen loss. Compared with DCON, the HT1 treatment exhibited substantial metabolic regulation. Typically, rapid accumulation of lactic acid lowered pH, thereby effectively suppressing protease activity, shortening the window for protein hydrolysis, and reducing nitrogen loss. In addition, metabolic flux shifted from amino acid degradation pathways toward organic acid synthesis and energy metabolism pathways, as manifested by the upregulation of organic acid synthesis, lipid metabolism, and some secondary metabolic pathways. Cluster analysis and PLS‐DA further indicated that the metabolic profile in the HT1 treatment was highly similar to that of the CON treatment, and diverged most from that of the DCON treatment, indicating that inoculation with LAB suppressed undesirable microbial populations via acidification and reshaped metabolic networks, reversing the disease‐driven metabolic shifts [78]. This mechanism aligns with the patterns previously reported: “rapid LAB establishment–improved substrate utilization–reshaping of metabolic pathways [55, 81, 82].”

Unlike other related studies, the present study systematically unveils the coupling mechanism in a disease context: disease–microbial changes–metabolic alterations–protein degradation. LAB inoculation can significantly reduce NH_3_–N levels and improve fermentation quality in healthy forage materials [83]. The present study further shows that, under disease conditions, LAB can reprogram metabolism to restore healthy metabolic features, a finding that extends the boundary of LAB applications. The present study further shows that, under disease conditions, LAB can reprogram metabolism to restore healthy metabolic features, a finding that extends the boundary of LAB applications. However, in this study, changes in metabolic flux depended mainly on estimates from metabolomics and relative bacterial abundance, and lacked direct verification of carbon/nitrogen flow via methods such as stable isotope tracing.

## Conclusion

Inoculation with *L. plantarum* HT1 resulted in a rapid decline in pH through homolactic fermentation, shortened the proteolysis window and suppressed undesirable microbial populations. Metabolic flux shifted from amino acid degradation toward organic acid and energy metabolism, thereby remodeling the metabolic network and reducing nitrogen losses. Future studies should simultaneously detect pathogen types and mycotoxins to achieve a more holistic understanding of the disease–toxin–silage quality relationship.

## Data Availability

Sequestration data for microorganisms were stored in NMDC (https://nmdc.cn/) with BioProject accession number NMDC40091587.
